# Toward Dual-Target Glycomimetics against Two Bacterial Lectins to Fight *Pseudomonas aeruginosa*–*Burkholderia cenocepacia* Infections: A Biophysical Study

**DOI:** 10.1021/acs.jmedchem.5c00405

**Published:** 2025-04-25

**Authors:** Giulia Antonini, Mario Fares, Dirk Hauck, Patrycja Mała, Emilie Gillon, Laura Belvisi, Anna Bernardi, Alexander Titz, Annabelle Varrot, Sarah Mazzotta

**Affiliations:** † Dipartimento di Chimica, Università degli Studi di Milano, Via Golgi 19, 20133 Milan, Italy; ‡ Helmholtz Institute for Pharmaceutical Research Saarland (HIPS), Helmholtz Centre for Infection Research, D-66123 Saarbrücken, Germany; § Deutsches Zentrum für Infektionsforschung (DZIF), Standort Hannover-Braunschweig, D-38124 Braunschweig, Germany; ∥ Department of Chemistry, PharmaScienceHub (PSH), Saarland University, D-66123 Saarbrücken, Germany; ⊥ CERMAV, Univ. Grenoble Alpes, CNRS, 38000 Grenoble, France

## Abstract

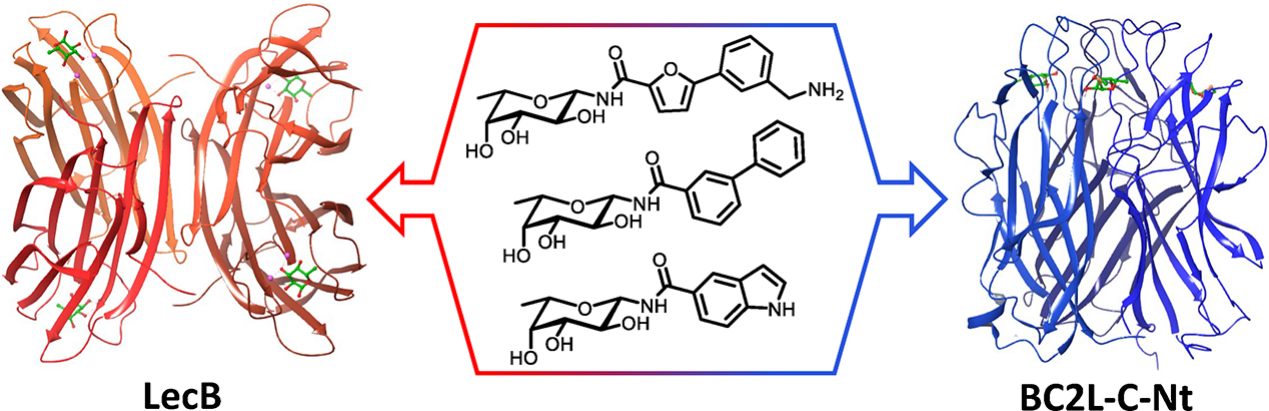

Chronic lung infections caused by *Pseudomonas aeruginosa* and *Burkholderia cenocepacia* pose a severe threat to immunocompromised patients, particularly those with cystic fibrosis. These pathogens often infect the respiratory tract, and available treatments are limited due to antibiotic resistance. Targeting bacterial lectins involved in biofilm formation and host–pathogen interactions represents a promising therapeutic strategy. In this study, we evaluate the potential of synthetic fucosylamides as inhibitors of the two lectins LecB (*P. aeruginosa*) and BC2L-C-Nt (*B. cenocepacia*). Using a suite of biophysical assays, we assessed their binding affinities, identifying three β-fucosylamides as promising dual-target ligands, while crystallography studies revealed the atomic basis of these ligands to interact with both bacterial lectins. The emerged classes of compounds represent a solid starting point for the necessary hit-to-lead optimization for future dual inhibitors aiming at the treatment of coinfections with these two bacterial pathogens.

## 1 Introduction

Antimicrobial resistance, especially from Gram-negative bacteria, is becoming a significant global challenge and urges for new treatments.^1,2^ Complex interactions between bacteria within the host significantly affect the progression of various diseases and further complicate treatment. Bacterial coinfections with multiple bacterial pathogens are often associated with synergistic effects that modulate the immune response and deteriorate the clinical outcome.^3^ These coinfections usually manifest as an enhancement of an existing infection process, especially when the immune system is compromised.^4,5^ The lung has a large exposed surface and is therefore highly sensitive to microbial colonizations. In cystic fibrosis (CF) patients with weakened defense mechanisms, airway infections are the primary cause of morbidity and mortality.^6^

*Pseudomonas aeruginosa* is among the most frequently isolated pathogens from the respiratory tract in CF patients.^7^ It is often found to be associated with other bacteria, such as *Burkholderia cenocepacia* and *Staphylococcus aureus*. In particular, the concomitant presence of *P. aeruginosa* and *B. cenocepacia* significantly worsens disease progression, especially in CF patients, leading to lung failure and premature death. *In vitro* and *in vivo* studies demonstrated a synergistic cooperation of these two species in biofilm formation, a major mechanism of antimicrobial resistance, persistence, and chronic colonization.^8−10^ Furthermore, coinfection with both species resulted in an increased immune response, deduced from increased levels of inflammatory cytokines and chemokines in lung tissue.^8^*B. cenocepacia* also affects the host innate immune system promoting *P. aeruginosa* persistence.^8,11,12^

Antimicrobial resistance, which is prevalent in these bacteria, especially in *P. aeruginosa*, often limits the use of standard-of-care antibiotics and implies the search for novel treatments.^13^ In the case of coinfections, the presence of different resistances in the different pathogens further complicates the situation. If effective antibiotic combinations are available to fight both coinfecting species, these are often at the cost of increased side effects. Therefore, the identification of novel molecules that can simultaneously hinder polymicrobial infections is appealing. To this end, a dual-target strategy to create drugs for two different targets in two species is a promising approach. Further, the use of a single drug can reduce costs of clinical development and unintended side effects, likely resulting in better safety profiles.^14^

Both *P. aeruginosa* and *B. cenocepacia* employ lectins to adhere to host tissue and form biofilms.^13,15^ Thus, bacterial lectins represent promising targets to develop antiadhesive agents against both bacteria. These agents could interfere with biofilm formation and prevent infection spread by blocking bacterial adhesion to the host glycoconjugates. They could be active alone to restore the efficacy of the immune response or supplemented with antibiotic therapies.^16,17^

Two soluble lectins are expressed by *P. aeruginosa*, LecA and LecB, and secreted and associated with the outer membrane of the bacteria. *In vitro*, both proteins were shown to be important for biofilm formation and LecA was reported as a factor for the invasion of host cells.^18−20^ In addition, the role of both lectins in murine infection experiments revealed their importance in pathogenesis.^21^ LecA adopts a β-sandwich fold with two curved β-sheets formed by four antiparallel β-strands with one binding site per subunit found at each corner.^22^ LecA recognizes nonreducing end α-d-galactosides present on glycosphingolipids through one calcium ion coordinating the O3 and O4 hydroxy groups. LecB folds as a nine stranded antiparallel β-sandwich with a Greek key motif and two β-sheets composed of four and five strands, respectively. The five stranded hydrophobic curved β-sheet is involved in the oligomerization, forming first dimers by head-to-tail association and then tetramers by antiparallel association and sheet extension of the dimers.^23^ Each subunit presents a binding site at its apex with two calcium ions essential for the recognition of α-l-fucosides with submicromolar affinity (methyl α-l-fucoside: *K*_D_ = 0.43 μM).^24^ LecB also recognizes d-mannosides but with lower affinity. Several variants of LecB are found in clinical and environmental isolates which display different fine specificities and affinities for fuco- or mannosylated ligands.^25^

*B. cenocepacia* expresses four soluble lectins: BC2L-A to -D. Besides the ability to bind host glycoconjugates and mediate bacterial cell adhesion, these lectins cooperate in biofilm matrix development and can potentially link quorum sensing with biofilm formation.^15,26^ They all present a LecB-like domain, while BC2L-B to -D also present an additional *N*-terminal domain. BC2L-A shows an exclusive specificity for d-mannose with an uncommonly high affinity for this monosaccharide (*K*_D_ (ITC) = 2.8 μM).^27^ BC2L-C is a hexamer that displays a double sugar specificity: the LecB-like calcium-dependent *C*-terminal dimeric domain is specific for heptose/mannose, while the *N*-terminal trimeric domain BC2L-C-Nt is l-fucose-specific and binds histo-blood group oligosaccharides, such as the H-type 1 antigen.^28^ In contrast to LecB, BC2L-C-Nt does not depend on Ca(II) ions for binding. It displays low affinity for its ligands with *K*_D_s in the mM range (*K*_D_ (ITC) = 2700 μM) for methyl α-l-fucoside, reaching the μM range (25 μM) for the H-type 1 trisaccharide.^29,30^

The development of glycomimetic inhibitors for these lectins is therefore of high interest as a novel approach to fight bacterial infection and resistance.^16,17^ Recently, our groups have separately reported the synthesis and biophysical evaluation of different libraries of fucosylamide-based glycomimetics able to interact either with LecB or BC2L-C-Nt.^31−33^

For LecB, mannose/fucose hybrids were initially developed as *C*-glycosidic inhibitors with high potency against both prevalent LecB variants from the two strains PAO1 and PA14^25,34−37^ when equipped with amide and sulfonamide substituents at position 6 following mannose numbering. Thermodynamic *K*_D_s ranged from 290 nM to 1.3 μM for sulfonamides and from 2.6 to 3.1 μM for the best amides. These compounds even showed inhibition of *P. aeruginosa* biofilm formation *in vitro*, while the equally potent LecB ligand methyl α-l-fucoside failed. Furthermore, good ADME parameters (metabolic stability in plasma and liver microsomes, solubility), no observed toxicity, and oral bioavailability in mice were demonstrated for the two best sulfonamide derivatives.

**Figure 1:**
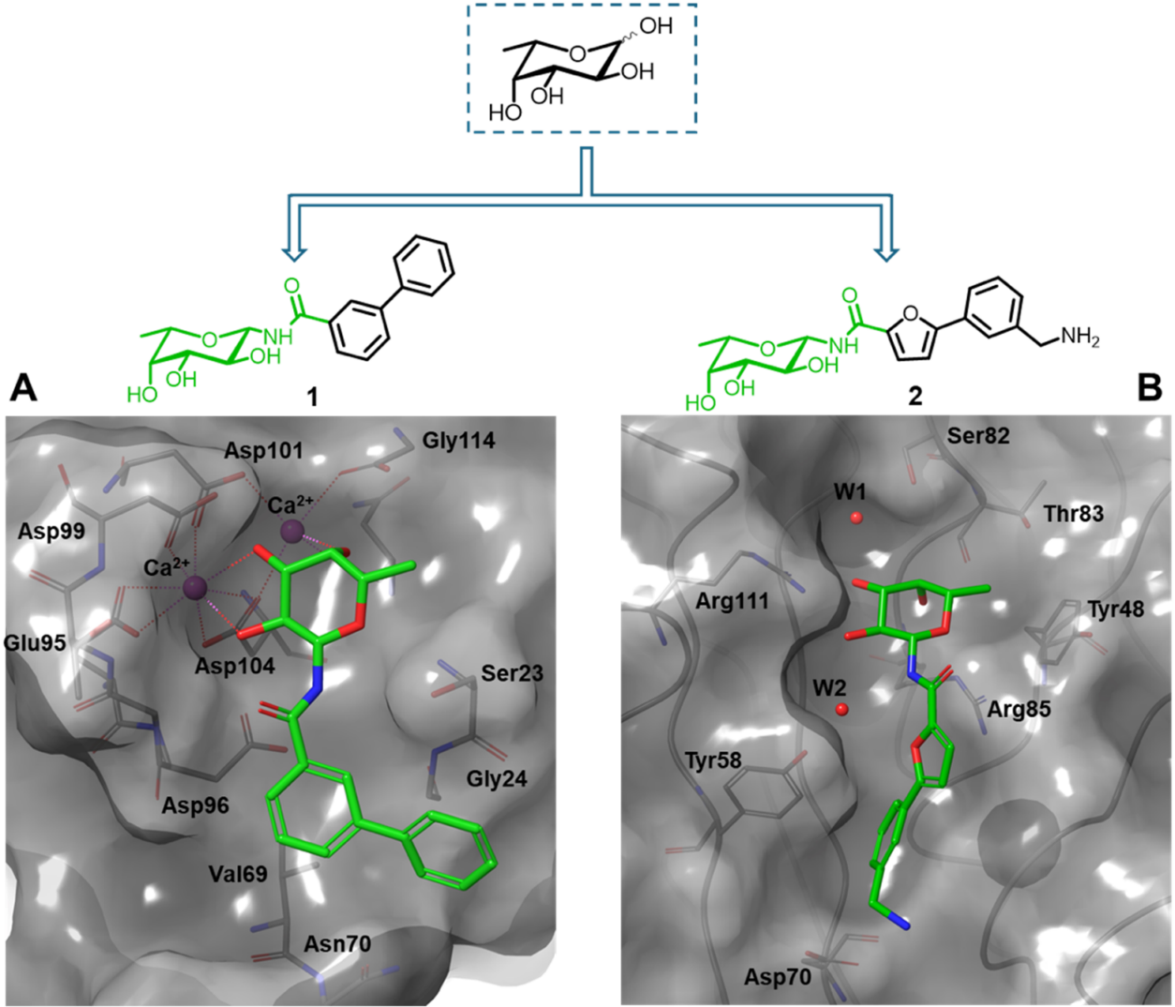
Crystal structures of β-fucosylamides in complex with *P. aeruginosa* and *B. cenocepacia* lectins: (A) **1** in complex with LecB (PDB: 8AIY)^31^ and (B) **2** in complex with BC2L-C-Nt (PDB: 8BRO).^32^

The possibility of shifting those amide and sulfonamide substituents by the extension or removal of the linker between the carbohydrate ring and those functional groups was also investigated. While an extension in the mannose series led to strongly reduced affinities for LecB, e.g., in 7-amido/sulfonamido mannoheptose (IC_50_s > 180 μM) compared to the mannose analogs (IC_50_s 3.4 and 34 μM), a shortening of this linker with direct attachment to the ring in the fucose series unexpectedly boosted the affinity of the resulting *N*-fucosylamide glycomimetics to 85–272 nM.^31,38^ The directly comparable cinnamide had an IC_50_ of 4.2 μM in the fucose/mannose hybrid series and 302 nM when shortened by one methylene group in the fucosylamide series, the best derivative for LecB was meta-biphenyl-carboxamide (**1**) with an IC_50_ of 85 nM (Figure 1A). Anomeric fucosylamides were reported as α-anomers before and were identified as moderate LecB inhibitors.^39^ The difference in binding affinity was quantified by ITC for β-fucosyl benzamide and its α-anomer, revealing *K*_D_s of 195 nM and 2.3 μM, respectively. This >10-fold loss of affinity for the α-anomer results from the fact that the substituent is mostly solvent exposed in the α series, while in the β-linked analogues, attractive interactions between amide and its substituents with LecB are observed by X-ray crystallography (Figure 1A). In fact, two amide orientations are seen in the cocrystal structure of the LecB tetramer with the biphenyl derivative **1**. Its phenyl aglycon establishes lipophilic contacts with Gly24 and Val69, and the distal phenyl ring with Asn70.

At the same time, a set of β-*C*- and β-*N*-fucosides was designed, synthesized, and evaluated as the antagonists of BC2L-C-Nt.^32,33^ They target a narrow cleft identified by *in silico* studies and formed at the interface of two monomers near the fucose-binding site.^40^ This cleft is not occupied by the known oligosaccharide ligands of the lectin, which all feature an α-fucoside, rather it extends in the direction of fucose β-anomeric position.^40,41^ A virtual screening campaign allowed the identification and experimental validation of fragments occupying the cleft, which is lined by the side chains of Tyr58, Arg85, and Asp70 (Figure 1B). Thus, the new β-fucosides contain the new identified fragments connected to the anomeric carbon through selected linkers of calculated length. Due to the stringent requirements of this narrow cleft (Figure 1B), some linkers were not well tolerated, but alkyne and amide linkers were predicted to fit. The resulting mimetics, synthesized and tested by SPR and/or ITC, resulted in an up to 10-fold affinity gain over methyl α-fucoside (*K*_D_ (ITC) = 2700 μM). In particular, β-fucosylamide **2** (Figure 1B) emerged as the most promising candidate, with a *K*_D_ value of 159 μM determined by ITC and a facile synthetic accessibility.^32^ The crystal structure of **2** in complex with BC2L-C-Nt revealed the key interactions driving the affinity for the target lectin and confirmed the docking predictions (Figure 1B). The sugar moiety that anchors the ligand to the binding site establishes hydrogen-bond interactions with Arg111, Thr83, and Arg85, and water-mediated interactions with Ser82 and Tyr58, while the C6 methyl group takes part in hydrophobic contacts with Tyr48.^32^ The aglycone fragment is involved in a T-shaped π-stacking interaction with Tyr58, and the terminal amino group establishes an H-bond and ionic interaction with Asp70. Finally, the amide NH participates in indirect hydrogen bonds with Tyr58, using a highly conserved water molecule (Figure 1B).

Given that both studies on LecB and BC2L-C-Nt independently revealed a comparable specificity for β-fucosylamides (Figure 1), we joined forces to systematically evaluate the affinity and interactions of glycomimetics for both lectins. Following our goal of identifying a potential dual-target ligand with a balanced affinity profile for both lectins, we aimed to provide a starting point for future structure optimization to inhibit coinfections of *P. aeruginosa* and *B. cenocepacia*. In this work, we present the affinity evaluation of fucose-based glycomimetics for the two targets, LecB and BC2L-C-Nt, followed by crystallographic studies to elucidate the molecular interactions of the identified hits with their targets. Three different classes of *N*-fucosylamides were identified as suitable for future optimization.

## 2 Results and Discussion

### 2.1 Biophysical Evaluation of Synthetic β-Fucosides’ Affinity for Both LecB and BC2L-C-Nt Lectins

Fucosylamides were screened against LecB from PAO1 using a previously established competitive binding assay^34^ based on fluorescence polarization. Here, we used the red-shifted fluorescent reporter fucoside **S3** carrying a Cy5 fluorophore (synthesis described in Scheme S1). For BC2L-C-Nt, a direct interaction SPR assay was established. The protein was immobilized by amide coupling on a sensor chip, and an apparent *K*_D_ for the inhibitors for BC2L-C-Nt was measured by injection of glycomimetics at increasing concentrations and affinity analysis. This assay has a good throughput and modest protein consumption but suffers from moderate sensitivity, given the low molecular weight of the ligands and their moderate affinity for the lectin. In order to appreciate its limits, we used the previously identified hit **2** (Table 1) for which data were already available from ITC (*K*_D_ = 159 μM)^32^ and SPR competition assay on a fucose chip (IC_50_ = 103 μM).^30^ The *K*_D_ of 185 μM determined by the direct interaction SPR assay is compatible with the previously reported data, which confirmed that this assay is able to recognize ligands in the same range of affinity as our current hit.

**Table 1:**
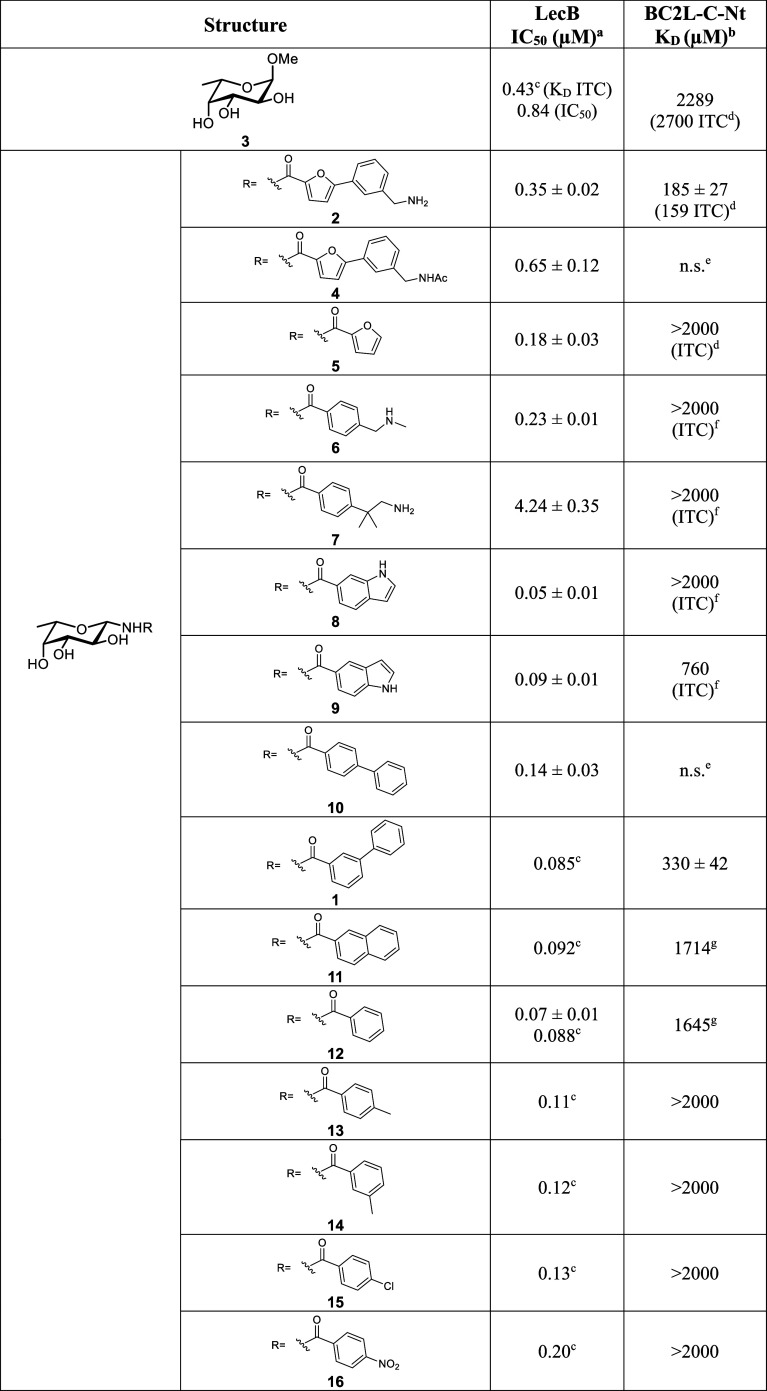

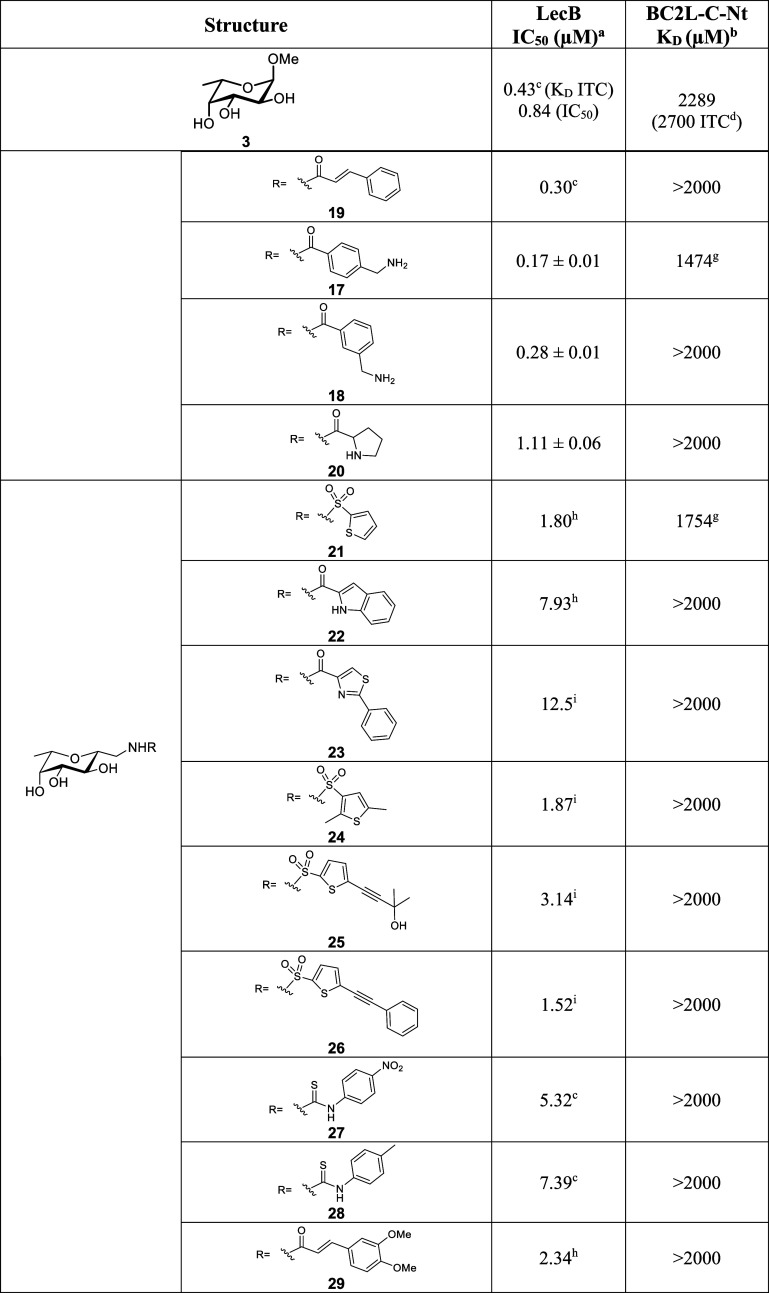
Affinity Evaluation of *C*- and *N*-Fucosides against LecB and BC2L-C-Nt Lectins

a Fluorescence polarization assay for LecB from strain PAO1.

b Direct interaction SPR assay with immobilized BC2L-C-Nt.

c From Mała et al.^31^

d From Mazzotta et al.^32^

e Not soluble at the required concentration.

f From Bermeo et al.^33^

g Only one experiment when *K*_D_ higher than 1000 μM.

h From Sommer et al.^36^

i From Sommer et al.^37^

**Figure 2:**
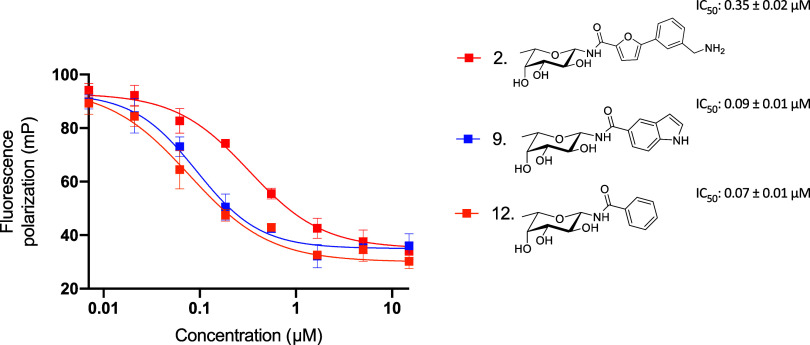
Competitive LecB binding assay showing the three fucosylamides, indolyl **9** and phenyl-furanoyl **2** and phenyl **12** as the standard. The IC_50_ and standard deviation values are obtained from three independent experiments. One representative replicate depicted in graph.

With these two assays in hand, we systematically evaluated a set of 29 β-fucosides to identify suitable starting points that may be developed as dual inhibitors. It can be noted at first glance that the intrinsic affinity of the two lectins for the core monosaccharide fucose differs by orders of magnitude (Table 1), which makes BC2L-C-Nt a more challenging target in this context. Indeed, all β-fucosylamides that we screened, including the BC2L-C hit **2**, displayed low μM to sub-μM IC_50_ values for LecB in the competitive binding assay (Table 1 and Figures 2 and S1). In particular, the indole derivatives **8** and **9** with IC_50_s of 50 and 90 nM, respectively, provide a new chemotype in the same range of affinity of the previously identified hits. On the contrary, only a few of the tested compounds bound to BC2L-C-Nt with μM affinity. In particular, **1** (SPR *K*_D_ = 330 μM) displayed an affinity comparable to that of **2** (Figure S2A). Furthermore, the 5-substituted indole **9** showed a *K*_D_ of 760 μM by ITC, whereas its regioisomer, the 6-substituted indole **8**, was 5-fold weaker (Figure S2B). Surprisingly, most other substituted benzamides and all tested *C*-fucosides (Table 1) did not display any significant binding to BC2L-C-Nt.

The presence of a methylene group at the anomeric position of the fucose core significantly reduced the affinity in BC2L-C-Nt as shown by the millimolar *K*_D_ values for these compounds (Table 1, compounds **21** to **29**. *K*_D_ from 1.75 to >2 mM). This effect could be explained with the structure of the binding site of *B. cenocepacia*’s lectin. The added methylene group could increase the flexibility of the ligands, making it harder for them to fit properly into the narrow cleft of the target binding site.

Among the direct β-fucosylamides, those with nonfused aromatic systems showed the highest affinities. Compounds with a single aromatic ring had reduced affinity, particularly with BC2L-C-Nt. The crystal structure of compound **2** in complex with BC2L-C-Nt (Figure 1B) reveals that the second aromatic ring interacts with the Tyr58 side chain, stabilizing the binding. This suggests that a more extended aromatic system is needed in BC2L-C-Nt to improve the affinity. Fused bicyclic compounds, such as **8**, **9**, and **11**, were less effective than nonfused compounds like **1**, **2**. This likely happens because the nonfused compounds, being more elongated, can better fit into the BC2L-C binding site and reach the Tyr58 side chain for a T-shaped interaction.

In contrast, the wider and more accessible binding site in LecB allows the accommodation of a variety of chemotypes, ranging from sulfonamides to aromatic amides.

Overall, our screening on LecB and BC2L-C-Nt yielded 3 fucosylamides as plausible candidates with a balanced affinity profile for future dual inhibitor development: furan derivative **2** (LecB IC_50_ 0.35 μM, BC2L-C-Nt *K*_D_ 185 μM), biphenyl derivative **1** (LecB IC_50_ 85 nM, BC2L-C-Nt *K*_D_ 330 μM) and indole derivative **9** (LecB IC_50_ 90 nM, BC2L-C-Nt *K*_D_ 760 μM). We therefore moved on to further characterize their interaction with the lectins via crystallographic studies.

### 2.2 Crystallographic Studies

To investigate the atomic-level interaction of the dual ligands and provide starting points for structure-based ligand optimization, we solved the missing crystal structures, i.e., the structures of LecB with dual ligands **2** and **9** and of BC2L-C-Nt with dual ligand **1**.

**Figure 3:**
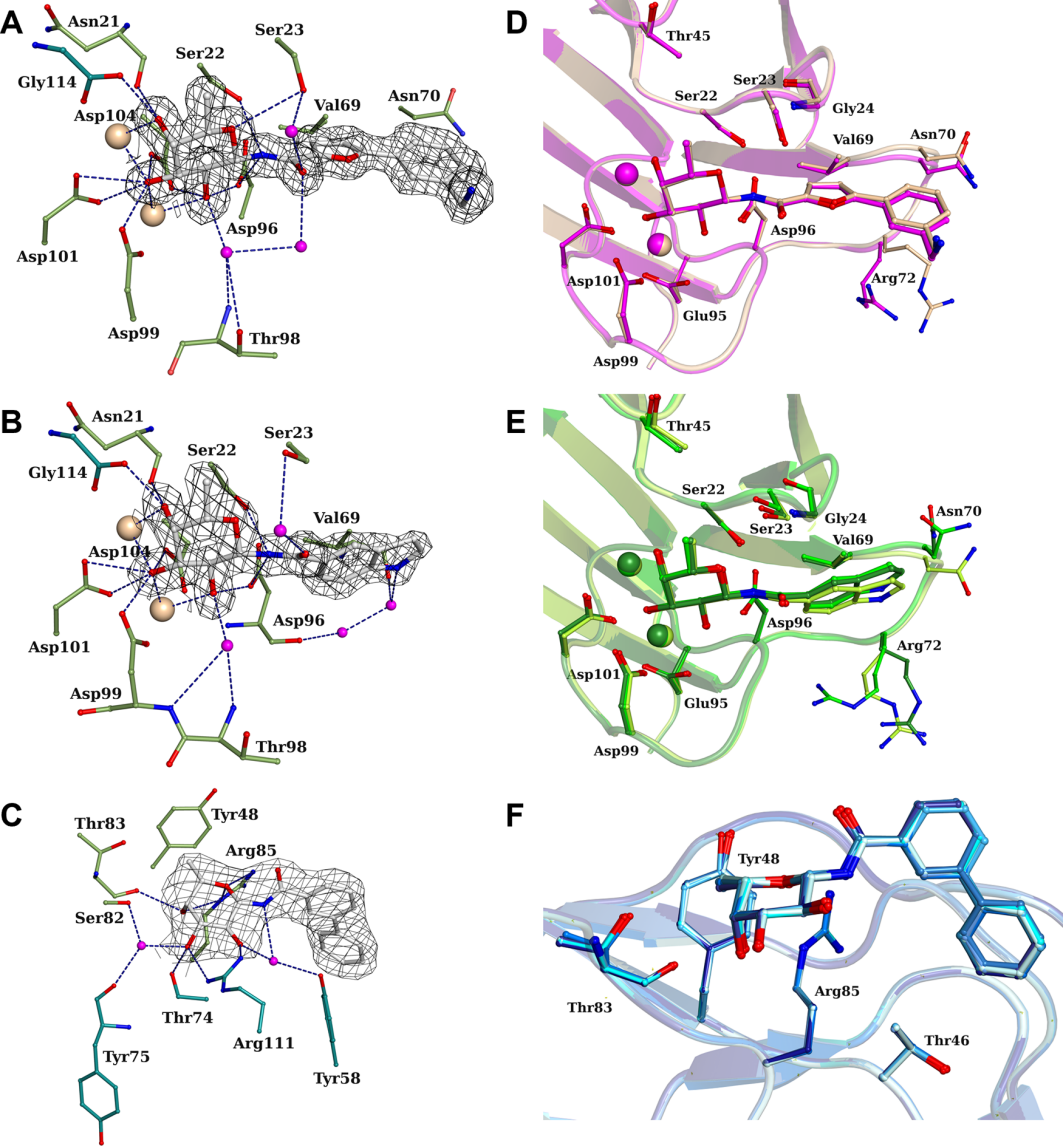
X-ray crystal structures of LecB or BC2L-C-Nt in complex with dual glycomimetics (PDB: 9G3K, 9G3L, and 9H0Q). Zoom on the interactions of LecB with **2** (A) or **9** (B) and BC2L-C-Nt with **1** (C), respectively. 2mFo-DFc electron density map contoured at the 1 sigma level is displayed around the ligand. Protein carbon atoms are colored according to protomers. Water molecules are depicted as purple spheres and calcium ions as light orange spheres. Direct or water-mediated hydrogen bonds with the ligand are shown in dashes. Overlay of binding sites for the different protomers of LecB binding **2** (D) or **9** (E) and BC2L-C-Nt binding **1** (F), respectively.

Co-crystals of LecB with the dual ligands **2** and **9** were obtained with a resolution of 1.55 and 1.74 Å, respectively (Figure 3A,B). Both complexes were solved in the *P*2_1_ space group with very similar unit cell, and one tetramer was observed in the asymmetric unit. Data collection and refinement statistics are summarized in Table S1.

In the complex of LecB with **2** (PDB: 9G3K), only the binding site in protomer D was found to be occupied by the fucosylamide ligand (Figures 3A,D and S3A). In the other three binding sites, calcium ions were clearly identified from the density map, but no ligand was detected. Instead, all of these binding sites present a sulfate ion originating from the crystallization condition. In protomer D, the fucose moiety coordinates to the calcium ions inside the carbohydrate binding site as reported previously for other fucoside/LecB complexes.^23,36^ This coordination involves the three hydroxy groups, OH2, OH3 and OH4, while the methyl group at position 6 is directed toward Ser23 and Thr45 (Figure 3D), forming hydrophobic interactions with them. The anomeric amide nitrogen forms direct hydrogen bonds with Ser22 and Asp96, while its oxygen makes water-mediated interactions with Ser23 and Thr98 (Figures 3A,D and S3). The aglycone part is oriented so that the furan oxygen and the terminal benzylamine point toward the solvent. The orientation of the furan ring allows for hydrophobic interactions with Gly24 and Val69. The phenyl ring conformation is imposed from the crystal contacts (Figure 3D): it is stacked between the side chain of Asn70 from one symmetric protomer and the main chain of the β strand containing Ser41 from another symmetric protomer on the other side (Figure S3C). The terminal benzylamine of **2** displays a direct H-bond and a water-mediated interaction with the main chain oxygen of Ser41 and Val69 from this last symmetrical protomer. In solution, the aglycone ring could adopt other conformations and stack on the LecB surface to optimize the interactions in particular of the amine.

In the complexes of LecB and **9** (PDB: 9G3L), all binding sites are occupied by fucosylamides (Figure 3B,E). In particular, in protomers B–D, the electron density can be attributed to the indole derivative **9**, while unexpectedly, in protomer A, the observed electron density corresponds to **2** (Figures S3B and S4). The observed mixed complex results from inadvertently transferring a **2** cocrystal in the cryosolution containing **9**, resulting in soaking that compound. The amide **2** is observed only in the binding site where its conformation is imposed by crystal contacts and presents the same interactions as described in Figures 3D and S3. The fucose scaffold of indole derivative **9** maintains a consistent position and coordination with calcium ions across all binding sites (Figure 3E). Notably, the indole fragment is oriented such that its NH group establishes water-mediated interactions primarily with the backbone carbonyl groups of Asp96 and Val69 (Figure 3B). It is stacked against Gly24 (Figure 3E) and presents slight variations in orientation, which seem related to the conformation of the side chain of Asn70 and resulting van der Waals interactions.

**Figure 4:**
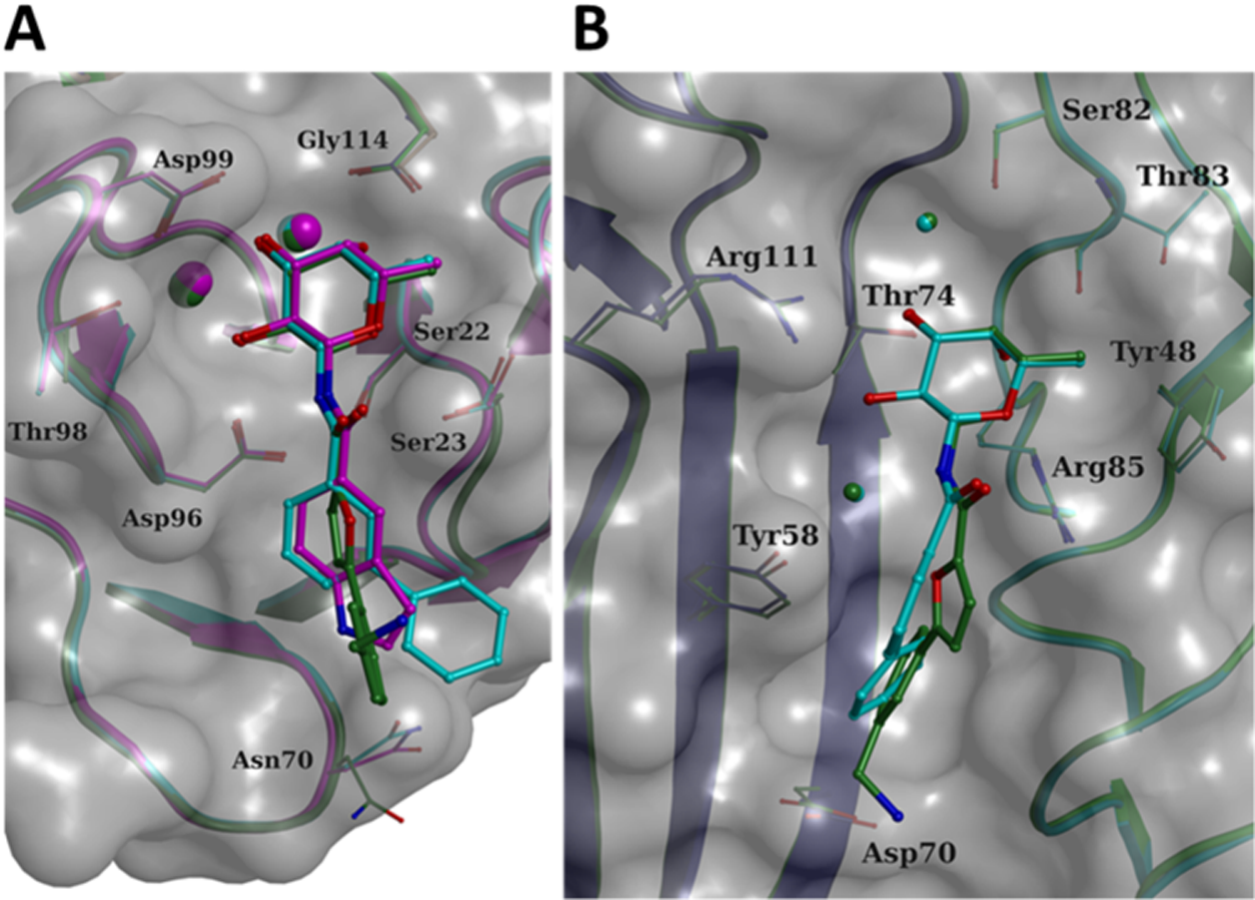
(A) Overlay of glycomimetics **1** (light blue), **2** (green), and **9** (purple) (PDB: 8AIY, 9G3K and 9G3L) in a LecB binding site. The two calcium ions are depicted as spheres. (B) Overlay of glycomimetics **1** (light blue) and **2** (green) (PDB: 9H0Q and 8BRO) in a BC2L-C-Nt binding site. Two conserved water molecules are depicted as spheres.

When comparing the new LecB complexes with the known LecB-biphenylamide **1** complex (PDB entry 8AIY, Figure 1), the overall protein structure remains clearly constant with the root-mean-square deviation (rmsd) ranging from 0.2 to 0.3 Å for the matched Cα carbons. Only minor differences in the orientation of the side chain are observed for some residues in the environment of the binding site, namely Asn70 and Arg72, which are solvent exposed and sometimes disordered. Regarding the fucoside residues, it is evident that the position of the sugar core is strongly maintained and constrained by its coordination to the two calcium ions (Figure 4A). While **1** presented several conformations for the aglycone, mainly as a results of crystal contacts, only one binding mode is observed for **2** and **9**. The benzene ring of **9** (purple in Figure 4A) and the first one of the biphenyl fragment of **1** (light blue in Figure 4A) almost overlap, while the furan ring in **2** (green in Figure 4A) is rotated by around 40° and does not lie in the same plane (Figure 4A).

To solve the structure of BC2L-C-Nt in complex with **1** new cocrystallization conditions were found, consisting of 17% Peg10K, 100 mM sodium acetate, and 100 mM Bis-Tris at pH 5.5. The structure could be solved at 2.55 Å in space group H32 with 10 molecules in the asymmetric unit comprising 3 trimers and one protomer along the 3-fold axis where the trimer is obtained by applying symmetry. All molecules present the same overall structure with a rmsd between 0.094 and 0.21 Å. All interactions with fucose are conserved and involve Arg111, Thr74, Tyr58, and Tyr75 from one protomer and Ser82, Thr83, Arg85, and Tyr48 from the other protomer, forming the binding site (PDB: 9H0Q, Figure 3C,F). Electron density is clearly observed for the aglycone in all protomers, while in some protomers one of the two structural waters could not be modeled (Figure S5). The two benzene rings of **1** bind in the narrow cleft of BC2L-C-Nt through hydrophobic and van der Waals interactions. The two aromatic rings of **1** in the complex are not coplanar but form a dihedral angle of about 28°. This orientation allows the distal phenyl ring to burrow into the crevice, while the vicinal ring interacts with Tyr58 in a T-shaped π-stacking. Considering that, both, the benzamide **12** and the naphthylamide **11** (Table 1) have negligible affinity for BC2L-C-Nt, the hydrophobic interaction of the distal phenyl ring of **1** in the deepest section of the binding site may represent a major contribution to the binding affinity of this ligand (Figure 4B).

**Figure 5:**
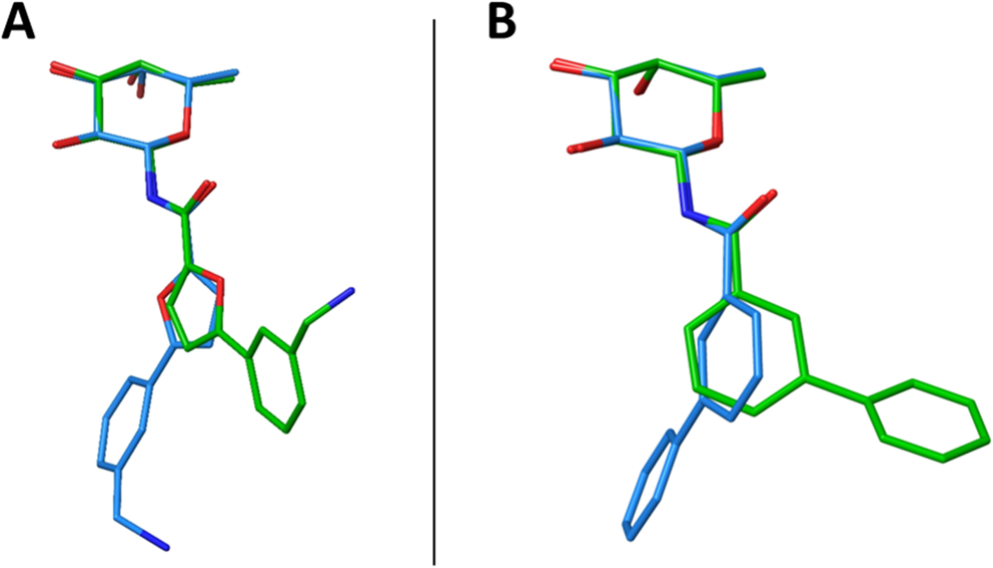
Overlap of ligand **2** (A) and **1** (B) in the binding sites of BC2L-C-Nt (light blue, PDB: 8BRO and 9H0Q) and LecB (green, PDB: 9G3K and 8AIY). The superposition was performed based on the fucose atoms, as the fucose core has the same conformation in both lectins.

Finally, comparing the complexes of **2** with LecB (PDB: 9G3K) and with BC2L-C-Nt (PDB: 8BRO), we observed that the aglycone is rotated approximately by 180° around the bond connecting the amide to the furan ring (O–C–C_fur_–O_fur_ of −179° in the BC2L-C-Nt complex and −4° in the LecB complex) and that the oxygen atom of the furan ring is directed toward the protein in BC2L-C-Nt, while it is solvent exposed in LecB (Figure 5A). The amide linking the fucose core to the aglycone portion adopts a similar orientation in both crystal structures. It assumes an antiperiplanar conformation relative to the anomeric proton (H_anomeric_–C_anomeric_–N–H angle of approximately 145°), a feature previously observed in other BC2L-C-Nt-fucosylamide complexes.^32,33^ Clearly, the amide conformation seems to be preserved regardless of the lectin, suggesting that it is preferred and serves as a stable and fixed characteristic of the ligands.

The amide maintains the same conformation also in both crystals of ligand **1** with BC2L-C-Nt (PDB: 9H0Q) and LecB (PDB: 8AIY), while rotation of the biphenyl moiety is observed in the two complexes (Figure 5B). In particular, the analysis of the dihedral angle between the amide and the biphenyl group (O–C–C_Ph_–C_*o-*Ph_) revealed a rotation of approximately 130°, with values of −160° in the BC2L-C-Nt complex and around −31° in the LecB complex. These differences are partly dictated by the different shapes of the binding sites for the two lectins. In BC2L-C-Nt, the binding site is found at the interface between two protomers and is more buried and extended than the site in LecB, which is shallower and solvent exposed (Figure 4). The flexibility brought by the phenyl-amide bond allows the different molecules to adapt to each lectin binding site and optimize notably hydrophobic or stacking interaction with the aromatic rings of the aglycon.

## 3 Conclusions

Our work is the first systematic biophysical evaluation of glycomimetics aiming at the simultaneous inhibition of two lectins from two bacterial pathogens. Here, we focused on two structurally unrelated lectins that were found to bind structurally similar inhibitors, i.e., β-fucosylamides.

LecB from *P. aeruginosa* binds most compounds of this class in the nM range, whereas BC2L-C-Nt from *B. cenocepacia* has a more stringent specificity toward the aglycone of this class and additionally only has μM affinity for the best derivatives. Thus, the challenge ahead lies in a balanced affinity profile allowing efficient binding of both lectins. We have identified three classes of most promising derivatives, i.e., biphenyl **1**, indolyl **9**, and phenyl-furanoyl **2** and have obtained high-resolution X-ray crystal structures for all three in complex with both lectins. These data now provide the starting point for hit-to-lead optimization in our laboratories. We are aiming at potent inhibitors of both lectins that might serve as efficient treatments for difficult-to-treat coinfections with *P. aeruginosa* and *B. cenocepacia*.

## 4 Material and Methods

The synthesis and characterization of compounds was previously described.^31−33,36,37^ The purity is >95% by HPLC-UV.

### 4.1 LecB Recombinant Expression and Purification

*P. aeruginosa* LecB (from strain PAO1) was expressed and purified as outlined previously.^42^ In summary, *Escherichia coli* BL21(DE3) containing the LecB-expressing plasmid pET25-paIIL were cultured in 1 L of LB medium supplemented with ampicillin (100 μg mL^–1^) at 37 °C and agitation at 180 rpm to an OD_600_ of 0.5–0.6. Protein expression was induced by adding IPTG (0.5 mM final concentration), and the bacteria were then incubated for 4 h at 30 °C and 180 rpm. Cells were then harvested by centrifugation (3000*g*, 10 min), and the resulting pellet was washed with PBS. Subsequently, the cells were suspended in 25 mL of TBS/Ca^2+^ solution (20 mM Tris, 137 mM NaCl, 2.6 mM KCl at pH 7.4, supplemented with 100 μM CaCl_2_), containing PMSF (1 mM) and lysozyme (0.4 mg mL^–1^), and disrupted on ice using a sonicator (5 cycles of 10 s). Cellular debris was eliminated through centrifugation (10 min, 10000*g*), and the supernatant was loaded to a column packed with mannosylated sepharose CL-6B.^43^ After the column was washed with TBS/Ca^2+^, LecB was eluted by adding 100 mM d-mannose to the buffer. The eluate was dialyzed against distilled water, followed by the lyophilization of the protein. The protein was reconstituted in TBS/Ca^2+^ prior to use, and its concentration was determined by UV spectroscopy at 280 nm, using a molar extinction coefficient of 6 990 M^–1^ cm^–1^ obtained by analyzing the amino acid sequence of LecB_PAO1_ (GenBank: AAG06749.1) on Protparam.^44^

### 4.2 BC2L-C-Nt Recombinant Expression and Purification

Expression was performed in *E. coli* BL21star (DE3) with the plasmid pCold-TF-TEV-BC2L-C-Nt in 1 L of LB medium overnight at 16 °C at 180 rpm after induction with 0.1 mM IPTG when OD_600 nm_ reached 0.7–0.8. The temperature was lowered from 37 to 16 °C when the OD_600 nm_ reached 0.4. After centrifugation of the cells at r.t. for 5 min at 5000*g*, the resulting pellet was weighed and each g of wet cell pellet was resuspended with 5 mL of buffer A (50 mM Tris-HCl pH 8.5, 100 mM NaCl). One μL (250 U) of DENARASE endonuclease (c-LEcta GMBH, Leipzig, Germany) was added for 10 min at r.t. with agitation using a rotating wheel. The cells were lysed using a one-shot table-top cell disruptor at a pressure of 1.9 MPa (Constant Systems Ltd.), and the lysate was centrifuged 30 min, 24000*g* at 4 °C. The resulting supernatant was filtered through a 0.45 μm polyethersulfone (PES) syringe filter and then loaded on a HisTrap fast flow (FF) column (Cytiva) equilibrated with buffer A. Unbound proteins were washed with buffer A prior elution with 20 column volumes (CV) and a gradient of 0–500 mM imidazole. Fractions containing the protein were pooled after examination on 12% SDS-PAGE gel. Imidazole was removed using a PD10 desalting column (Cytiva) and buffer A; TEV cleavage was performed overnight at 19 °C using a protein/TEV ratio of 1/50 in the presence of 0.5 mM EDTA and 0.25 mM TCEP and a protein concentration of at least 1 mg mL^–1^. The sample was loaded on a HisTrap column, and the cleaved fraction was collected in the flow through and concentrated using a centrifugal device with a cutoff of 3 kDa. It was then submitted to size exclusion chromatography using an ENrich SEC 70 10 × 300 column pre-equilibrated with 20 mM Tris-HCl pH 7.0 and 100 mM NaCl using an NGC system (Bio-Rad Ltd.). After analysis on 15% SDS-PAGE, pure trimer fractions were pooled, concentrated when necessary, and stored at 4 °C. The concentration was determined by UV spectroscopy at 280 nm, using a molar extinction coefficient of 19940 M^–1^ cm^–1^.

### 4.3 LecB Competitive Binding Assay Using Fluorescence Polarization

The assay was conducted as per Hauck et al. with some modifications.^34^ Briefly, 10 μL of a stock solution of LecB (150 nM) and red-shifted Cy5-Fuc reporter ligand **S3** (20 nM) in TBS/Ca^2+^ with 0.3% DMSO were added to 10 μL of serially diluted (15 μM to 117 nM) testing compounds in the same buffer in triplicates, into black 384-well microtiter plates (Greiner Bio-One, Germany, cat no 781900). The microtiter plates were then centrifuged at 1500*g* for 1 min, covered with a plastic foil (EASYseal, cat no. 676001, Greiner Bio-One) and incubated for 5 h at r.t. under the exclusion of light in a black humidified container under rocking conditions. After incubation, fluorescence intensity was measured with a PheraStar FS microtiter plate reader (BMG Labtech GmbH, Germany) at excitation 590 nm and emission 675 nm. Data analysis was conducted using MARS Data Analysis Software (BMG Labtech GmbH, Germany) after subtracting blank values (75 nM LecB in TBS/Ca^2+^ with 0.3% DMSO) from the samples. Fluorescence polarization was calculated, and data fitting was done following the four-parameter variable slope model. The experiment was independently repeated three times, and data were averaged and displayed using GraphPad PRISM version 9.

### 4.4 Synthesis of Cy5-Labeled α-l-Fucoside S3

Sulfo-Cy5-carboxylic acid (**S1**, 23.7 mg, 36.1 μmol) was dissolved in DMF (400 μL) and EDC·HCl (10.4 mg, 54.3 μmol) was added at r.t. The mixture was stirred for 5 min, triethylamine (7.6 μL, 54.1 μmol) was added, and then the mixture was cooled to 0 °C. 2-Aminoethyl-α-l-fucopyranoside^34^ (**S2**, 15.0 mg, 72.4 μmol) dissolved in 300 μL DMF was added, and the reaction was allowed to warm to r.t. and stirring was continued for 5 days. Additional coupling reagent did not change only approximately 50% product formation. Volatiles were removed under reduced pressure. The residue was purified by flash chromatography on silica using isocratic elution with EtOAc/EtOH/H_2_O = 9/9/2 and the title compound (5.5 mg, 18%) was obtained as a blue solid.

ESI-MS calcd for [C_41_H_55_N_3_O_12_S_2_–H]^−^ 844.3, found 845.0. ^1^H NMR (500 MHz, H_2_O) δ 8.14 (dt, *J* = 12.5, 8.8 Hz, 2H), 8.00–7.90 (m, 4H), 7.48–7.39 (m, 2H), 6.65 (t, *J* = 12.4 Hz, 1H), 6.50–6.22 (m, 1H), 4.95 (d, *J* = 3.8 Hz, 1H, Fuc-H1), 4.25–4.13 (m, 4H), 4.07 (q, *J* = 6.6 Hz, 1H, Fuc-H5), 3.93 (dd, *J* = 10.3, 3.3 Hz, 1H, Fuc-H3), 3.87 (dd, *J* = 10.5, 4.0 Hz, 1H, Fuc-H2), 3.85 (d, *J* = 3.1 Hz, 1H, Fuc-H4), 3.80 (ddd, *J* = 10.5, 7.0, 3.8 Hz, 1H, CH_2_), 3.61 (ddd, *J* = 10.2, 6.1, 3.9 Hz, 1H, CH_2_), 3.49 (ddd, *J* = 14.4, 7.2, 3.9 Hz, 1H, CH_2_), 3.39 (ddd, *J* = 14.4, 6.1, 4.0 Hz, 1H, CH_2_), 2.39–2.32 (m, 3H), 1.97–1.86 (m, 2H), 1.79–1.69 (m, 14H), 1.51–1.38 (m, 6H), 1.26 (d, *J* = 6.6 Hz, 3H, Fuc-H6). ^13^C NMR (126 MHz, H_2_O, extracted from ^1^H,^13^C HSQC) δ: 154.10, 126.45, 125.67, 119.73, 110.82, 103.95, 98.32 (Fuc-C1), 71.76 (Fuc-C4), 69.58 (Fuc-C3), 68.01 (Fuc-C2), 66.61 (Fuc-C5), 66.45 (CH_2_), 43.64 (CH_2_), 39.26 (CH_2_), 38.95 (CH_2_), 35.51 (CH_2_), 26.76 (CH_3_), 25.36 (CH_2_), 15.36 (Fuc-C6), 11.76 (Et-CH_3_).

### 4.5 BC2L-C-Nt Affinity Assay Using SPR

Experiments were performed on BIACORE X100 or T200 instruments (GE Healthcare) at 25 °C in running buffer (10 mM Hepes (pH, 7.4), 150 mM NaCl, and 0.05% Tween 20). BC2L-C-Nt was immobilized onto CM5 chips (BIACORE) by following an amine coupling procedure. To this end, the chip was activated by three injections of an NHS/EDC mixture at 5 μL min^–1^ for 600 s, until a minimum of 1000 RU was observed on both channels. Then, BC2L-C-Nt (0.25 mM) dissolved in 10 mM sodium acetate at pH 4.5 was injected onto channel 2 (contact time of 600 s, flow rate of 5 μL min^–1^), until a minimum of 3000 RU was observed for BC2L-C-Nt. Finally, both channels were inactivated by injecting a 1 M ethanolamine (pH 8.5) solution at 10 μL min^–1^ for 540 s, achieving over 2600 RU for channel 2.

The analytes were diluted in the running buffer (adding up to 5% DMSO when needed) at increasing concentrations (range: 15.65–2000 μM) and subjected to single- or multicycle affinity studies (200 s of association, 100 s of dissociation, flow rate 20 μL min^–1^). Injections of compounds at increasing concentrations onto the immobilized BC2L-C-Nt were followed by regeneration of the surface using 10 mM fucose in running buffer for 100 s at a flow rate of 20 μL min^–1^. Duplicates were performed for ligands with KD lower than 1000 μM. Binding affinity in terms of *K*_D_ was measured after subtracting the channel 1 reference (no immobilized protein) and subtracting a blank injection (running buffer with zero analyte concentration). Data evaluation and curve fitting were performed using the provided BIACORE X100 and T200 evaluation software.

### 4.6 Crystallization and Structure Determination

Crystallization was performed using the vapor diffusion method with hanging drops of 2 μL containing a 1/1 (v/v) ratio of protein/mother liquor at 20 °C in 24 wells VDX Plate with sealant (Hampton Research).

For the complex LecB-**2**, lyophilized LecB was dissolved in 20 mM Hepes pH 7.5 and 1 μM CaCl_2_ to 12.1 mg mL^–1^ and cocrystallized with 0.5 mM ligand after incubation at r.t.. Rod like crystals were obtained in a few days from solution 26 of the Structure Screen 2 (Molecular Dimensions, Calibre Scientific, U.K.) containing 30% PEG 5KMME, 200 mM ammonium sulfate, and 100 mM MES pH 6.5. Single crystals were directly mounted in a cryoloop and flash-frozen in liquid nitrogen. For the complex LecB-**9**, LecB was dissolved in Milli-Q water supplemented with 200 μM CaCl_2_ to 8.8 mg mL^–1^. Plate clusters appeared in 2 days in a solution containing 26% Peg6K, 1 M lithium chloride, and 100 mM sodium acetate pH 4.5. Single plates were cut from the cluster, transferred with a cryoloop, and soaked in a solution containing 32% Peg smear low (Molecular Dimensions), 50 mM sodium acetate pH 4.5, and 10 mM ligand for 3 min prior flash-freezing in liquid nitrogen. For BC2L-C-Nt-**1** complex, the protein at 5.2 mg mL^–1^ in 20 mM Hepes pH 7.5 and 100 mM NaCl was incubated with 1 mM ligand prior cocrystallization. Crystals were obtained in less than a week with a solution containing 17% Peg10K, 100 mM sodium acetate, and 100 mM Bis-Tris pH 5.5. The crystals were transferred for cryoprotection in a solution containing 7.6% Peg10K, 22.5% Peg smear low, 45 mM sodium acetate and Bis-Tris pH 5.5, and 3 mM **1**, mounted in a cryoloop and flash-frozen in liquid nitrogen.

Diffraction data were collected at 100 K at the synchrotron SOLEIL (Saint Aubin, France) on Proxima-2 beamline using an DECTRIS EIGER X 9M detector for LecB-**2** and BC2L-C-Nt-**1** complexes and on Proxima-1 beamline using an DECTRIS EIGER X 16M detector for the complex LecB-**9**. Data were processed using XDS^45^ and XDSme^46^ and all further steps were carried out using CCP4i, version 8.0.^47^

All structures were solved by molecular replacement using PHASER^48^ searching for 1 tetramer of LecB using the coordinates of PDB-ID 1GZT as model, and 3 trimers and 1 monomer using the coordinates of PDB-ID 2WQ4 and 6TID, respectively as models for BC2L-C-Nt. Restrained maximum likelihood refinement was performed using REFMAC 5.8 and local NCS restrains^49^ with manual rebuilding in Coot.^50^ For cross-validation analysis, 5% of the observations were set aside, while hydrogen atoms were added in their riding positions and used for geometry and structure-factor calculations. The ligand library was constructed with Acedrg.^51^ TLS refinement was also used for the BC2L-C-Nt-**1** complex where each protomer was defined as a group. The final coordinates were validated thanks to Molprobity and the wwPDB Validation server: http://wwpdb-validation.wwpdb.org(52) prior deposition with their corresponding structure factors in the Protein Data Bank.

## Data Availability

The crystallographic data are publicly available from the PDB website using the ID codes 9G3K and 9G3L for LecB in complex with **2** and **9**, and ID code 9H0Q for BC2L-CNt with **1**. Raw diffraction images and initial processing files are available on Zenodo under doi 10.5281/zenodo.12793535, 10.5281/zenodo.12794355, and 10.5281/zenodo.13912326, respectively.

## Supporting Information Available

Scheme for the synthesis of Cy5-labeled α-l-fucoside; competitive binding assay to LecB; SPR and ITC affinity assay on BC2L-C-Nt; crystallographic data; NMR spectra for Cy5-labeled fucoside S3; HPLC traces for ligands **1**, **2** and **9**. (PDF)
Molecular formula strings. (CSV)

## Abbreviations Used

*B. cenocepacia*: *Burkholderia cenocepacia*
CF: cystic fibrosis
ITC: isothermal titration calorimetry
*P. aeruginosa*: *Pseudomonas aeruginosa*
SPR: surface plasmon resonance
