# Supplementary material for: Toward Dual-Target Glycomimetics against Two Bacterial Lectins to Fight Pseudomonas aeruginosa–Burkholderia cenocepacia Infections: A Biophysical Study
Source: J Med Chem. 2025 Apr 25;68(9):9681–93. doi: 10.1021/acs.jmedchem.5c00405 (PMC12067436; doi:10.1021/acs.jmedchem.5c00405)

Supporting information

TITLE:

Towards dual-target glycomimetics against two bacterial lectins to fight *Pseudomonas aeruginosa-Burkholderia cenocepacia* infections: a biophysical study

----

AUTHORS:

Giulia Antonini[1], Mario Fares[2,3,4], Dirk Hauck[2,3,4], Patrycja Mała[2,3,4], Emilie Gillon[5], Laura Belvisi[1], Anna Bernardi[1], Alexander Titz[2,3,4*], Annabelle Varrot[5*] and Sarah Mazzotta[1*]

AFFILIATIONS:

[1]Dipartimento di Chimica, Università degli Studi di Milano, Via Golgi 19, 20133 Milan, Italy

[2]Helmholtz Institute for Pharmaceutical Research Saarland (HIPS), Helmholtz Centre for Infection Research, D-66123 Saarbrücken, Germany.

[3]Deutsches Zentrum für Infektionsforschung (DZIF), Standort Hannover-Braunschweig, D-38124 Braunschweig, Germany.

[4]Department of Chemistry, PharmaScienceHub (PSH), Saarland University, D-66123 Saarbrücken, Germany.

[5]Univ. Grenoble Alpes, CNRS, CERMAV, 38000 Grenoble, France

*Correspondence: alexander.titz@helmholtz-hzi.de; annabelle.varrot@cermav.cnrs.fr; sarah.mazzotta@unimi.it;

INDEX

1. Synthesis of Cy5-labelled α-L-fucoside 2

2. Competitive binding assay to LecB 2

3. SPR and ITC affinity assay on BC2L-C-Nt 3

4. Crystallographic studies 4

5. NMR spectra 8

6. HPLC traces for ligand 1, 2 and 9 11

1. Synthesis of Cy5-labelled α-L-fucoside

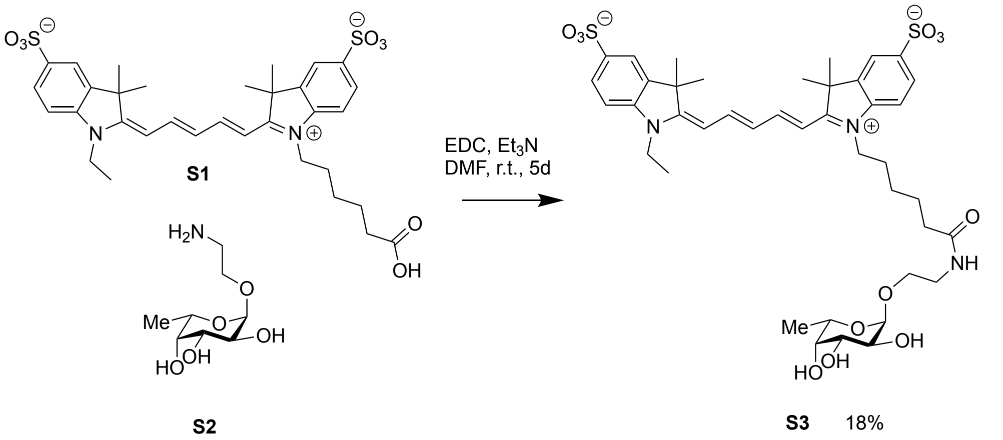

Scheme S1. Synthesis of Cy5-labelled α-L-fucoside as red-shifted fluorescent tracer for the competitive LecB binding assay.

2. Competitive binding assay to LecB

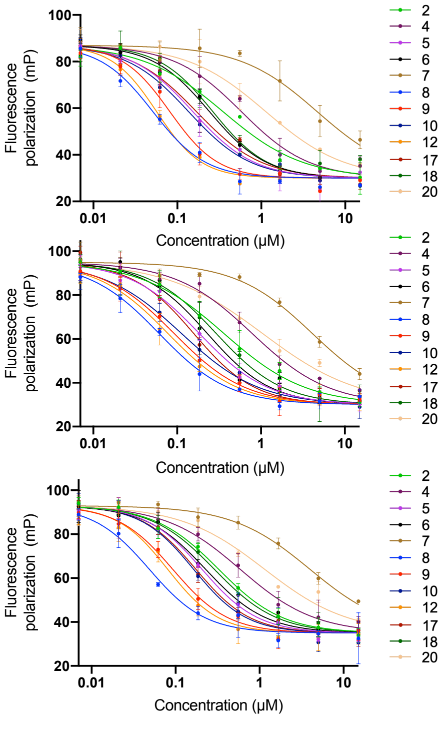

Figure S1. Complete data set of competitive binding assay to LecB for the dual inhibitor ß-fucosyl amides. Each graph represents an independent experiment of one titration of triplicates on one plate.

3. SPR and ITC affinity assay on BC2L-C-Nt

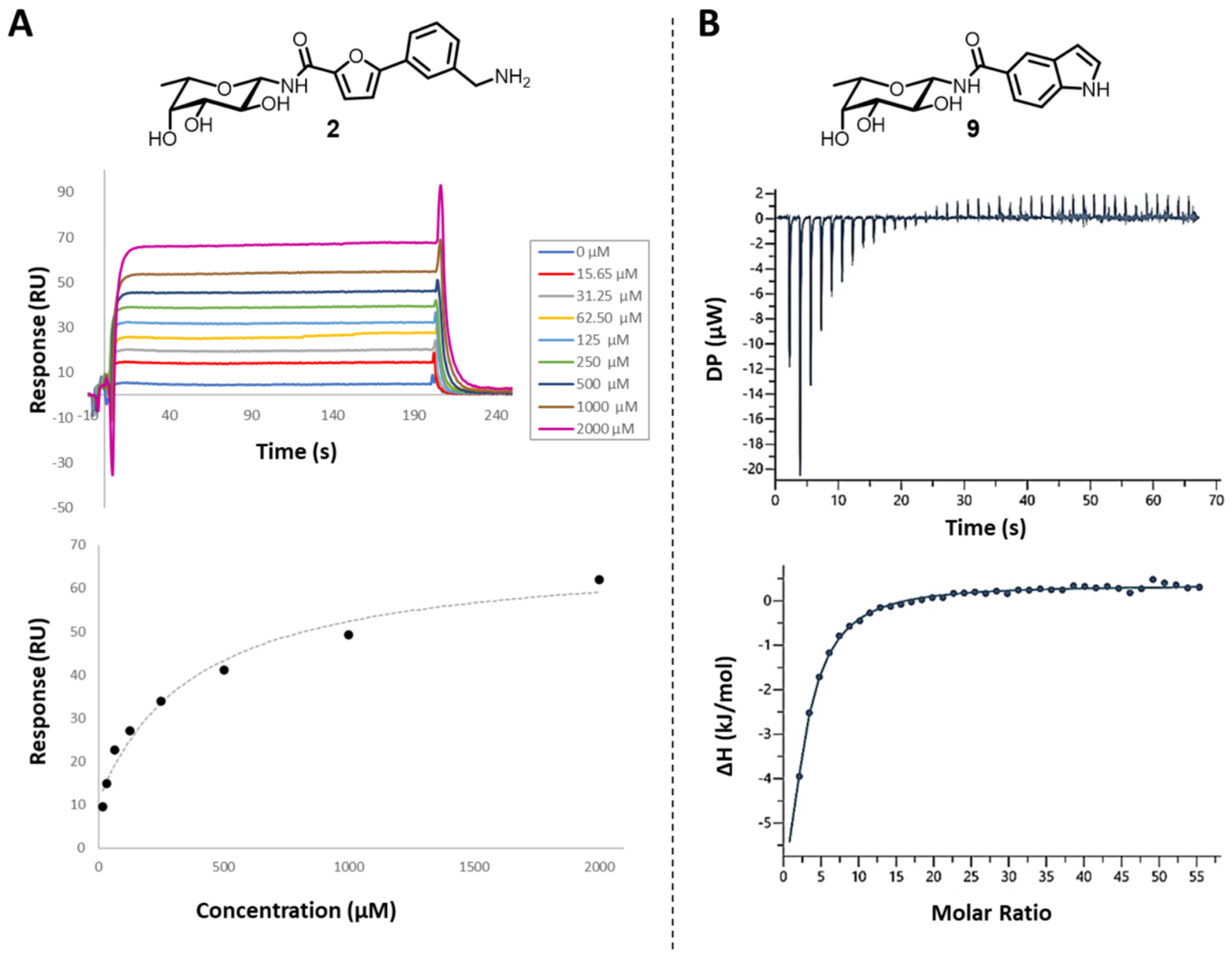

Figure S2. A) Sensorgram and affinity curve obtained from SPR analysis of **2** on chip functionalized with BC2L-C-Nt. B) Thermogram and titration curve obtained from ITC experiment with BC2L-C-Nt and **9.**

4. Crystallographic studies

Table S1: Data collection and refinement statistics

**Complex**	**LecB-2**	**LecB-9**	**BC2L-C-Nt-1**
Beamline	Soleil PX2	Soleil PX1	Soleil PX2
Wavelength (Å)	0.980112	0.978565	0.97856
Space group	$P2_1$	$P2_1$	H32
Cell parameters - a, b, c (Å)	50.35 80.17 52.41	51.02 80.16 52.43	169.51 169.51 344.04
α, β, γ (°)	90.00 109.36 90.00	90.00 109.61 90.00	90.00 90.00 120.00
Nb molecules in ASU	4	4	10
Resolution (Å)[a]	47.50-1.55 (1.58-1.55)	49.39-1.74 (1.77-1.74)	48.93-2.55 (2.62-2.55)
Number reflections[a]	268205 (12508)	212983 (9365)	721297 (54622)
Number of unique reflections[a]	56788 (2831)	40572 (1952)	62109 (4529)
$CC_{1/2}$ (%)[a]	99.8 (92.2)	99.4 (67.8)	99.9 (83.1)
R_{merge} (within I+/I-)[a]	0.048 (0.370)	0.140 (0.815)	0.091 (0.916)
R_{meas} (within I+/I-)[a]	0.060 (0.471)	0.172 (1.007)	0.099 (1.001)
R_{pim} (within I+/I-)[a]	0.036 (0.289)	0.099 (0.584)	0.029 (0.289)
Mean I/σ (I)[a]	15.5 (3.5)	7.6 (4.8)	18.6 (2.7)
Completeness (%)[a]	99.7 (99.7)	97.8 (80.8)	100 (100)
Multiplicity[a]	4.7 (4.4)	5.2 (4.8)	11.6 (12.1)
Wilson ***B***-factor ($Å^2$)	14	12.5	43.9
Resolution (Å)	47.50-1.55	49.39-1.74	48.93-2.55
No. reflections / free reflections	53908 / 3996	38474 / 2612	59045 / 3061
R_{work}/R_{free} (%)	13.6 / 16.8	14.7 / 18.8	17.9 / 22.2
R.m.s Bond lengths (Å)	0.014	0.014	0.014
Rmsd Bond angles (°)	1.834	1.692	1.889
Rmsd Chiral ($Å^3$)	0.106	0.099	0.087
Clashscore	2	2	3
Fo,Fc correlation	0.98	0.96	0.95
No. atoms / Bfac ($Å^2$)			
Protein	3386 / 15.4	3330 / 12.7	9599 / 54.5
Ligand	26 / 14.8	92 / 16	250 /42.7
Waters	590 / 28.9	571 / 24.2	377 /45.8
Ramachandran Allowed (%)	100	100	100
Favored	97.3	97.4	97.5
Outliers	0	0	0
PDB code	9G3K	9G3L	9H0Q

[a]Values in parenthesis refer to the highest-resolution shell.

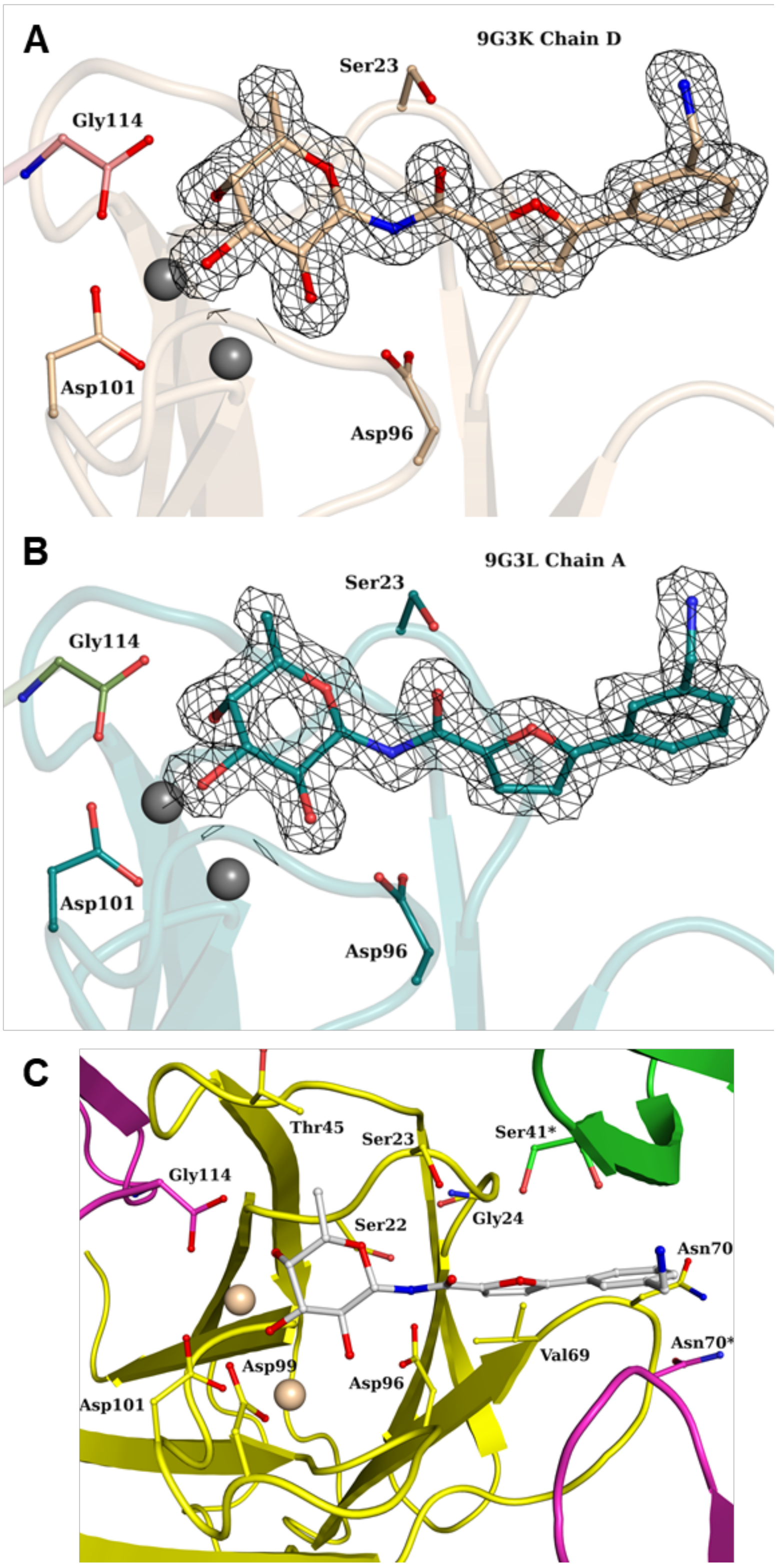

Figure S3. A-B) 2mFo-DFc electron density map contoured at 1 sigma level is displayed around **2** bound to LecB in PDB: 9G3K (0.44 eA^3) and PDB: 9G3L (0.48 eA^3), respectively. Protein carbon atoms are colored according to protomers. Calcium ions are displayed as grey spheres. C) Crystal contacts formed around aglycone of **2**. Coloring by protomers name and residues from symmetrical protomers are annotated with a *.

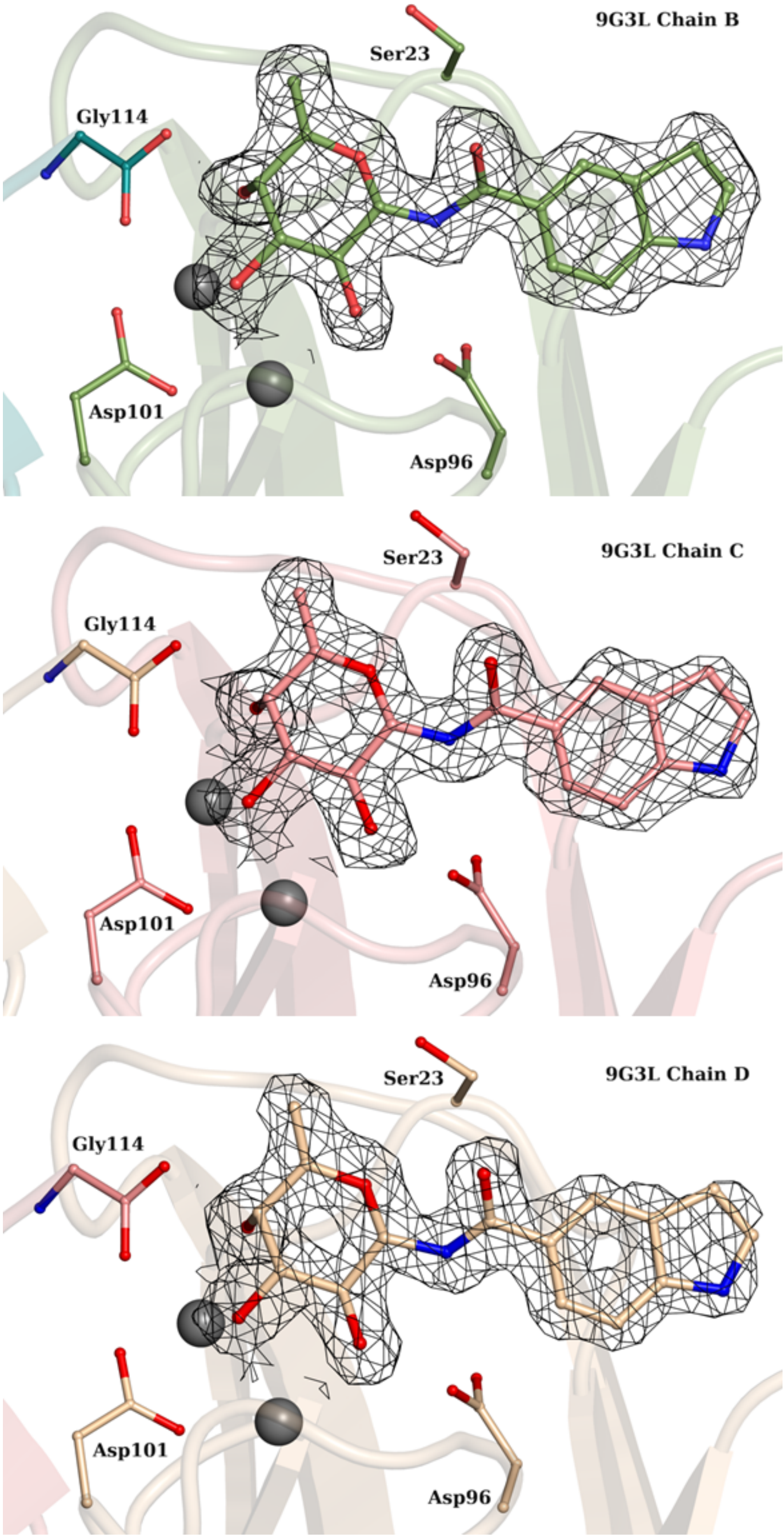

Figure S4. 2mFo-DFc electron density map contoured at 1 sigma level is displayed around **9** bound to LecB (PDB: 9G3L, 0.48 eA^3), respectively. Protein carbon atoms are colored according to protomers. Calcium ions are displayed as grey spheres.

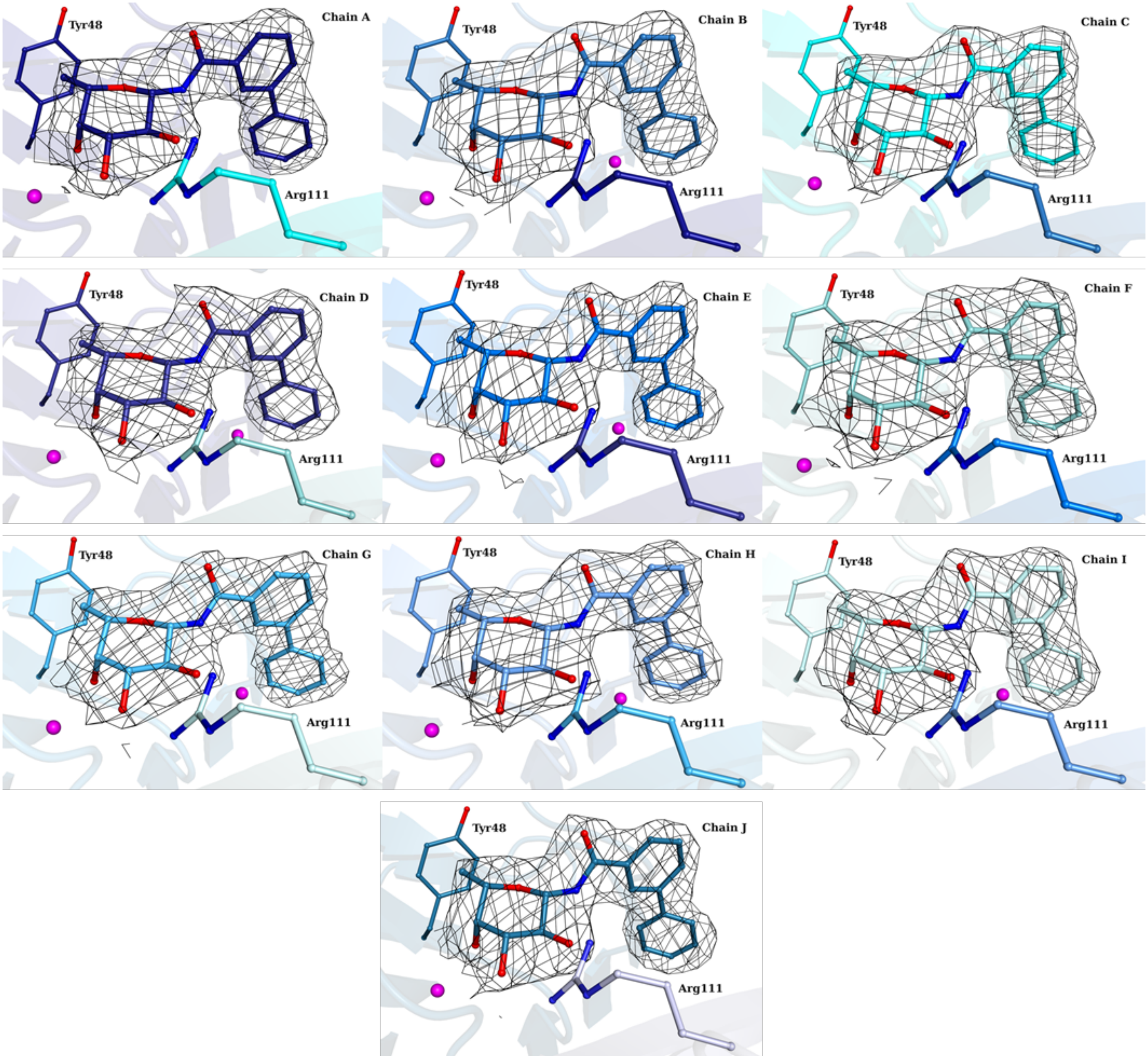

Figure S5. All binding sites of **1** in the asymmetric unit of the complex with BC2L-C-Nt. 2mFo-DFc electron density map contoured at 1 sigma level is displayed around **1** bound to BC2L-C-Nt (PDB: 9H0Q, 0.48 eA^3), respectively. Protein carbon atoms are colored according to protomers. Structural water molecules are displayed as purple spheres.

5. NMR spectra

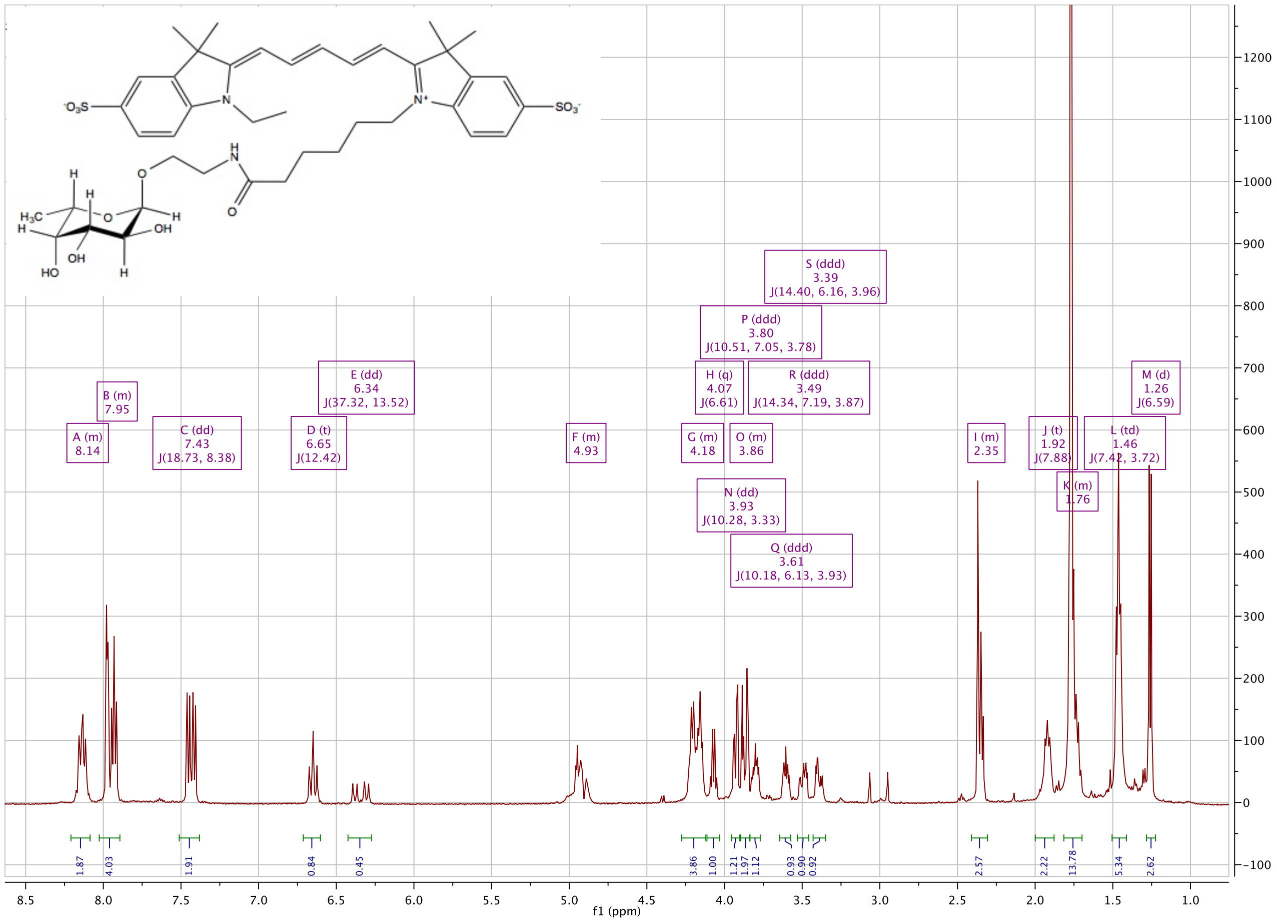

Figure S6: ^{1}H NMR of Cy5-labelled fucoside **S3**

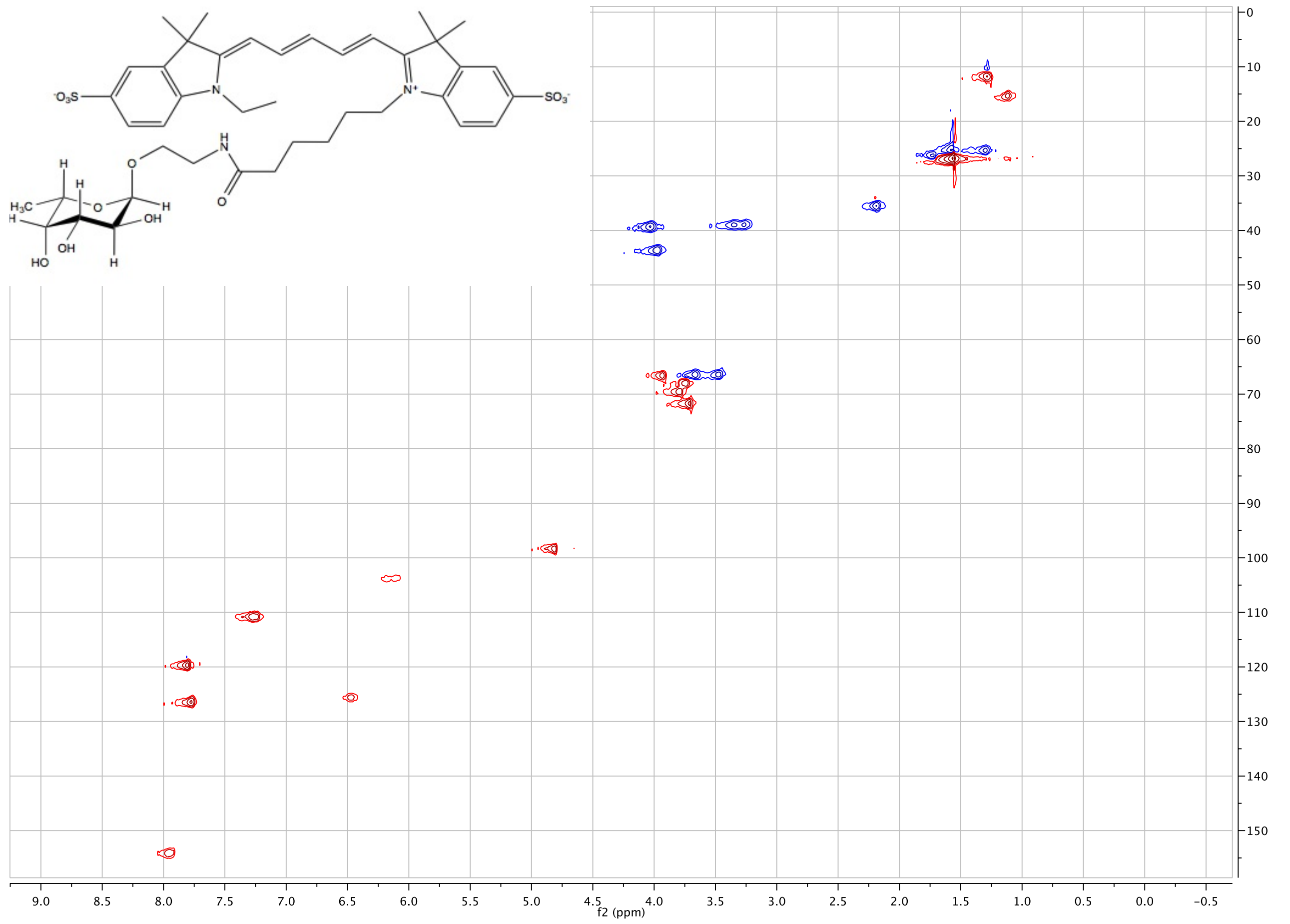

Figure S7: DEPT-edited 1H,13C-HSQC NMR of Cy5-labelled fucoside **S3**

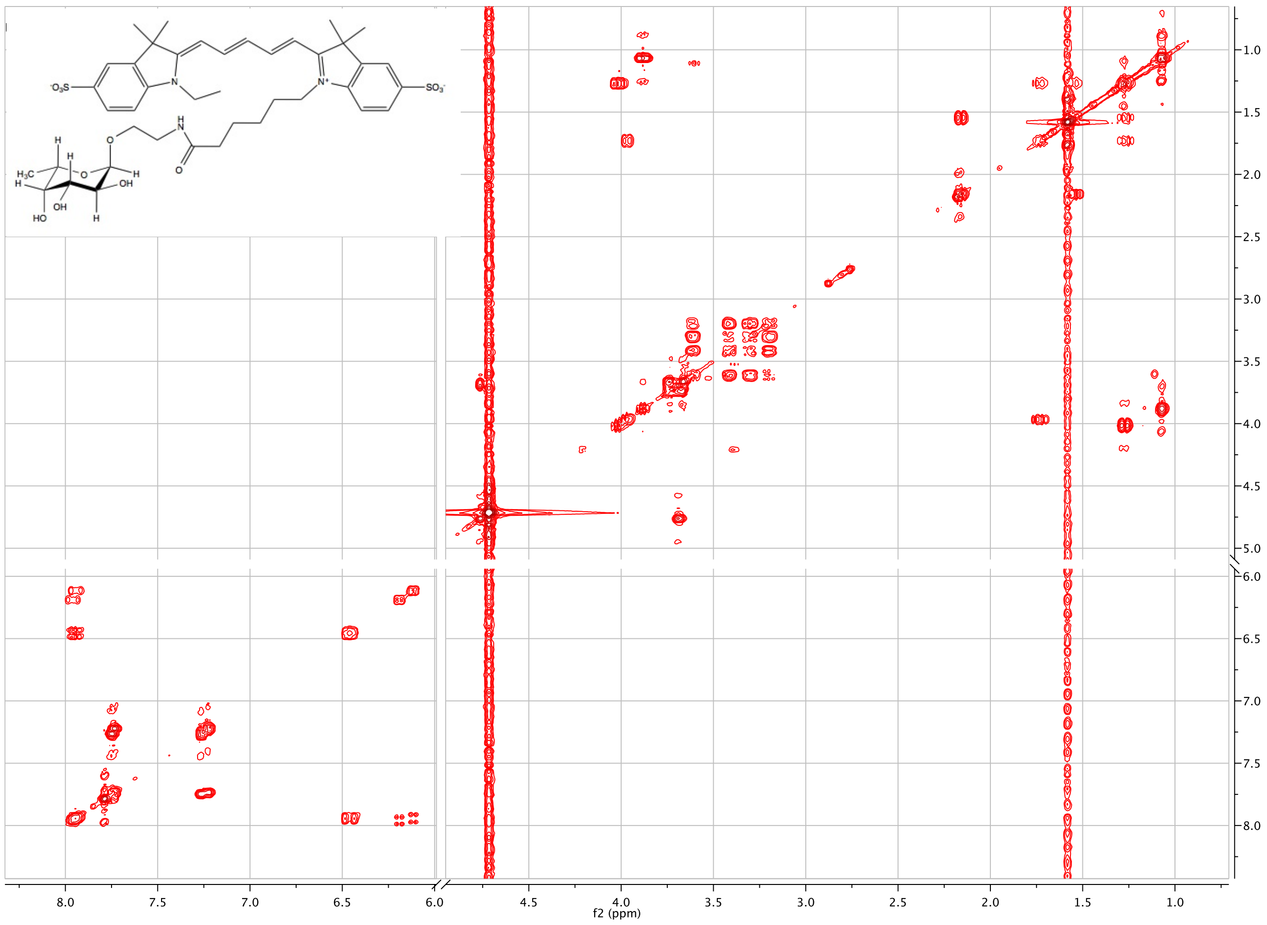

Figure S8: $^{1}H,^{1}H$-COSY NMR of Cy5-labelled fucoside **S3**

6. HPLC traces for ligand 1, 2 and 9

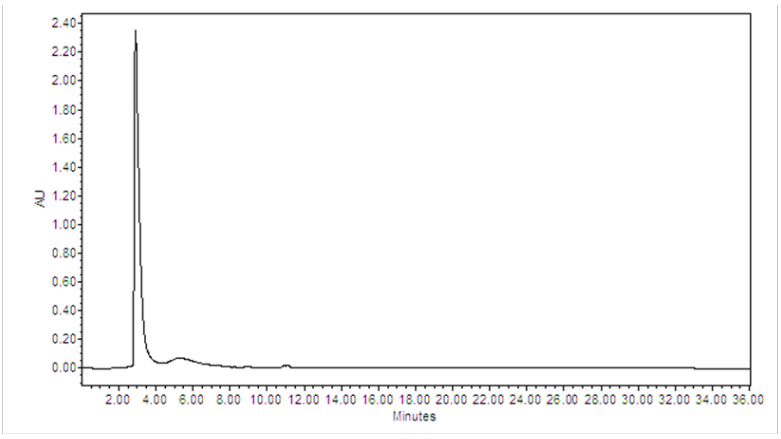

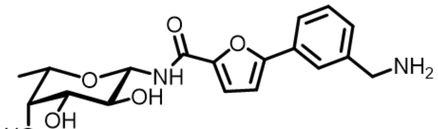

2

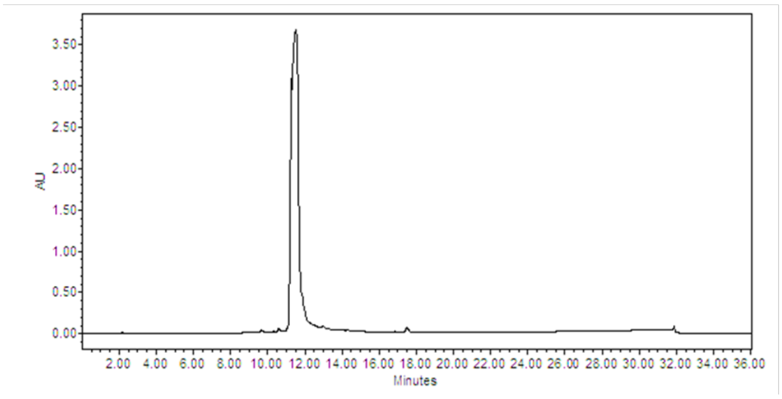

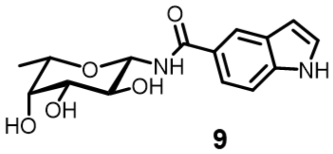

9

Generic Display Report

Analysis Info

		Acquisition Date	8/23/2024 5:31:19 PM
Analysis Name	D:\Data\0_CBCH\2024\Dirk\sonstige\Fimb_purity\PMA127_check_0824-Fimb_BD5_1_14784.d		
Method	14784.m	Operator	demo
Sample Name	PMA127_check_0824-Fimb	Instrument	amaZon SL
Comment			

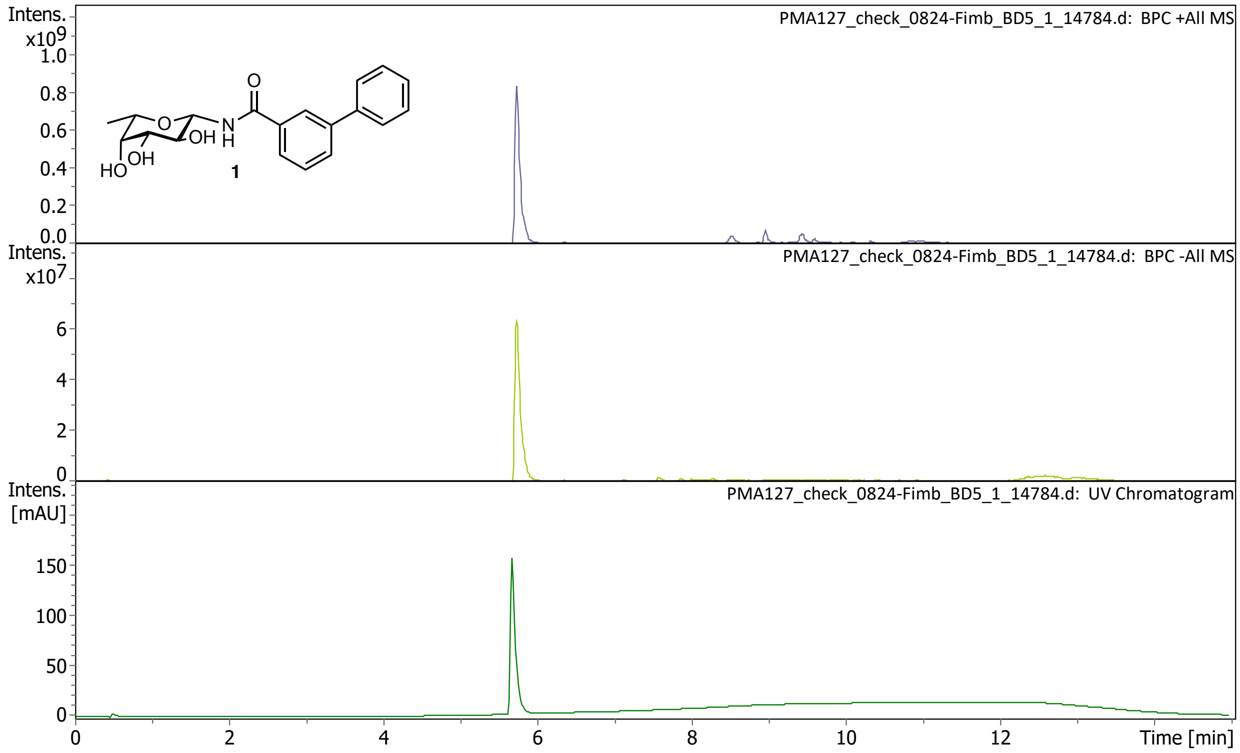

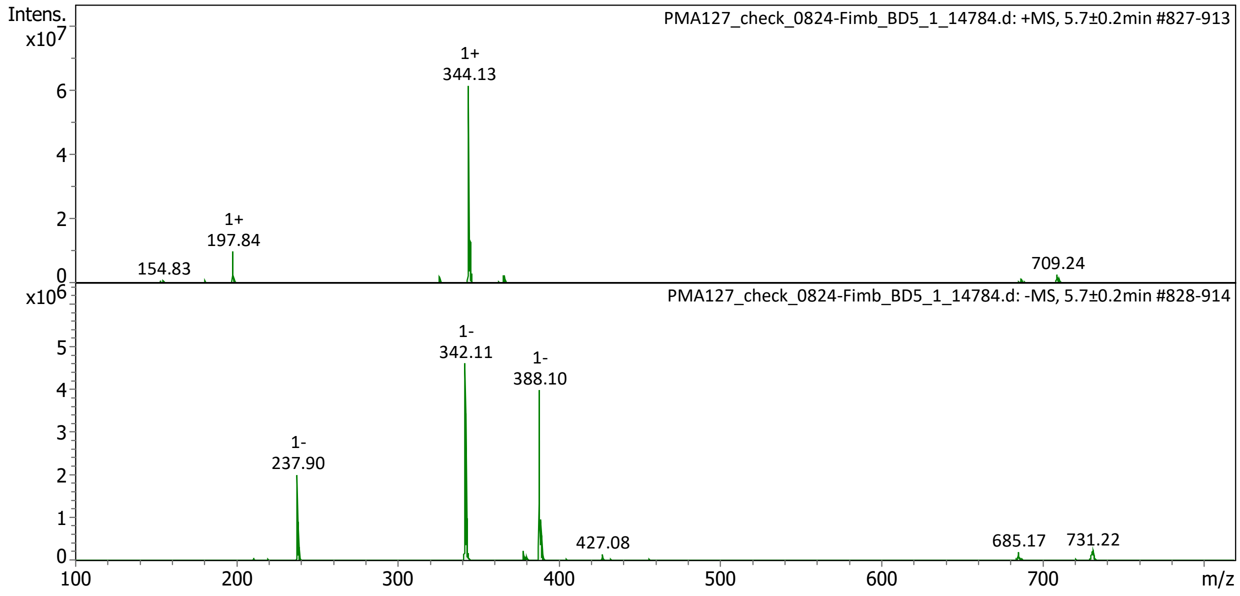

Supplement: Supplementary file 1 — jm5c00405_si_001.pdf [file jm5c00405_si_001.pdf]
